# PrEP as a “Hidden Message”: The Impact of Partner Dynamics on Oral PrEP Adherence Among Cisgender Women in Serodiscordant Relationships

**DOI:** 10.1007/s10461-025-05008-y

**Published:** 2026-01-23

**Authors:** Thupten D. Phuntsog, Jamila K. Stockman, K. Rivet Amico, Ryan Kofron, Sheldon Morris, Raphael J. Landovitz, Steffanie Strathdee, David J. Moore, Jill Blumenthal

**Affiliations:** 1 School of Public Health and Health Sciences, University of Massachusetts Amherst, Amherst, MA USA; 2 Department of Medicine, University of California San Diego, San Diego, CA 92103 USA; 3 Department of Health Behavior and Health Equity, School of Public Health, University of Michigan, Ann Arbor, MI USA; 4 UCLA Center for Clinical AIDS Research & Education, University of California Los Angeles, Los Angeles, CA USA; 5 Department of Psychiatry, University of California San Diego, San Diego, CA USA

**Keywords:** Pre-exposure prophylaxis, Cisgender women, Serodiscordant relationships, Adherence, Partner dynamics

## Abstract

**Background:**

Heterosexual sex accounts for 87% of new HIV diagnoses among cisgender women. We sought to explore the intersection of partner dynamics and oral preexposure prophylaxis (PrEP) adherence among cisgender women in heterosexual serodiscordant relationships.

**Methods:**

From June 2017-August 2018, we conducted semi-structured in-depth interviews using a social ecological model framework. Twenty cisgender women in serodiscordant relationships, who participated in a PrEP demonstration project to evaluate adherence and retention in San Diego and Los Angeles, participated in in-depth interviews. Interviews were audio-recorded and transcribed, and transcripts were analyzed using thematic analysis.

**Results:**

Among the 20 participants, the median age was 37.5 years (IQR 32, 48), with *n* = 6 (30%) identifying as Black and *n* = 5 (25%) as Hispanic. Sixty-five percent of women had protective drug levels based on real-time tenofovir-diphosphate drug level assays at the study visit prior to their interviews. Some partners played a significant role in PrEP adherence, often showing support by vocalizing encouragement and appreciation, offering reminders to take PrEP, and sometimes administering PrEP. Other partners were unsupportive and discouraged PrEP use, which may have hindered adherence. HIV and PrEP stigma were identified as potential barriers for women to take PrEP and disclose their PrEP use to others.

**Conclusions:**

Within the context of this PrEP demonstration project, partner dynamics impacted PrEP adherence for HIV-negative cisgender women in serodiscordant relationships. We urge further investigation of relationship dynamics and PrEP adherence and persistence specifically among US-based cisgender women.

## Background

When taken regularly, pre-exposure prophylaxis (PrEP) has been proven to be highly efficacious in preventing HIV [1, 2]. Despite its federal approval in 2012 [3], PrEP uptake has remained low among cisgender women in the United States [4] Cisgender women constitute nearly 20% of new HIV diagnoses each year [5], yet comprise less than 7% of PrEP users [6]. With heterosexual sex attributing to 87% of new HIV diagnoses among cisgender women and HIV being more efficiently transmitted from cisgender men to women [7, 8], it is critical to investigate PrEP use among heterosexual serodiscordant relationships in the U.S [9].

Serodiscordant, also referred to as serodifferent, relationships are defined as sexual relationships in which one individual is living with HIV and the other is HIV-negative [10]. In previous studies, the combination of antiretroviral therapy (ART) for the partner with HIV and PrEP for the partner without HIV has demonstrated the potential to notably reduce the risk of HIV transmission [11, 12]. However, research regarding serodiscordant relationships largely centers around men who have sex with men (MSM) and is mainly based in sub-Saharan Africa [13, 14]. Little is known about the viewpoints and experiences of HIV-negative cisgender women in US-based serodiscordant relationships, and given the increased risk for HIV exposure if the partner living with HIV is not consistently virally suppressed, serodiscordant couples are a key target population for HIV prevention [14–16].

Cisgender women in serodiscordant partnerships have reported a range of facilitators and barriers to PrEP use, including the need to reduce anxiety around HIV acquisition [17]. Challenges include PrEP- and HIV-related stigma and concerns around negative reactions from friends and family [18], and the role of relationship dynamics has been identified as influential to PrEP uptake and use [17, 19]. The complexities of PrEP adoption and engagement for women in the context of serodiscordant partnerships remain an important area of inquiry that could advise support services and programs.

Social ecological models (SEMs) have commonly been used to explain associations between social and structural factors, individual practices, the physical environment, and health [20]. By exploring perceived barriers and facilitators to PrEP adherence at individual, interpersonal, community, and societal levels in the context of a social ecological framework, we may be able to help frame the complex social and behavioral processes that affect medication adherence [21]. For cisgender women at risk of HIV infection through vaginal exposures, there are only two FDA-approved PrEP medications available: oral daily tenofovir disoproxil fumarate/emtricitabine (TDF/FTC) and bi-monthly injectable cabotegravir [22]. Despite advances in biomedical HIV-prevention leading to the availability of long-acting injectable PrEP in the US, barriers to PrEP implementation [23] and uptake contribute to low coverage among cisgender women [24]. Given that oral daily PrEP remains highly relevant for women in the US, we explored the experiences of women in serodiscordant relationships who received oral daily PrEP within a demonstration study in California.

We conducted a qualitative sub-study among HIV-negative cisgender women in serodiscordant relationships who participated in a larger longitudinal clinical trial that aimed to evaluate medication adherence and retention. We sought to examine perceived barriers and facilitators to their PrEP adherence and explore the impact of partnership dynamics and disclosure of PrEP use on PrEP adherence. Our ultimate goal was to better inform interventions and resources tailored specifically for cisgender women in serodiscordant relationships.

## Methods

### Parent Study

PrEP Adherence Enhancement Guided by iTAB and Drug Levels for Women (AEGiS) was a 48-week (with 60-week post-study visit) open-label single-arm longitudinal clinical trial that assessed medication adherence and retention among 136 at-risk women 18 years of age or older in Southern California. All participants received once-daily oral TDF/FTC through a comprehensive HIV PrEP prevention program, which employed daily, personalized, automated 2-way text messages and counseling support based on results from drug-level testing [25].

### Study Design and Participants

Only participants from the AEGiS parent study who gave permission to be contacted for other research and reported being in a serodiscordant relationship for at least 4 weeks were eligible to participate in this qualitative sub-study. During week 4 of the AEGiS study, participants at the Los Angeles and San Diego study sites were approached by study personnel to inquire about their interest in interviews to collect contextual data on PrEP adherence among women in serodiscordant relationships. Participants were enrolled based on their interest and availability to participate in an in-person interview. Convenience sampling continued until the planned sample size was reached. Guided by the SEM, we pre-specified domains at the individual, interpersonal, community, and structural levels for interview guide development and analysis, and we synthesized themes by level to reflect the ways the levels jointly shaped PrEP adherence.

### Data Collection

Twenty cisgender women who reported being in a serodiscordant relationship completed in-depth interviews. We identified 20 based on anticipated data adequacy [26] and previous literature suggesting this number is a reasonable expectation for coverage of a broad array of experiences within well-defined groups [27]. All interviews were conducted in English by a study co-investigator with several years of research and clinical experience in HIV prevention. A semi-structured qualitative interview guide was constructed to include open-ended questions and probes regarding key topics of interest framed within an SEM framework, including (1) experiences being in a relationship with an individual living with HIV and the dynamics within their relationship (individual, interpersonal); (2) motivations for and experiences taking PrEP (individual, interpersonal); (3) barriers and facilitators to PrEP adherence (individual, interpersonal, community, structural); (4) disclosure of their PrEP use and partner’s HIV status (interpersonal, community); and (5) opinions and recommendations regarding community, messaging, and access (community, structural). Critical elements, such as the sampling process, the organizing of materials gathered along thematic lines, and the reflexive, iterative nature of data collection, supported the data collection process. More specifically, the study team reflected on how her social position could shape rapport, questioning, and interpretation. During data collection, the interviewer maintained reflexive journals and held post-interview debriefs after several sessions to document how identity, assumptions, and emergent insights influenced probes. These notes led to minor refinements to the interview guide (e.g., adding probes, softening wording). During analysis, we drew on analytic memoing; discrepancies in coding were resolved in meetings that explicitly revisited positionality notes.

From June 2017 to August 2018, in-depth, confidential, audio-recorded interviews in English were conducted at the Los Angeles and San Diego study sites. Interviews took place between weeks 24 and 60 of the parent study, and each interview lasted approximately 45 min. Upon completion of data collection, one research personnel transcribed each interview using ExpressScribe software, which was then quality checked by another research personnel for accuracy. Participants received $30 compensation for their time and travel to the interview sites. Demographics and drug levels from dried blood spot (DBS) specimens were collected through the parent study at the visit preceding the in-depth interview, followed by extraction to describe the sub-study sample.

### Data Analysis

We conducted a hybrid inductive-deductive thematic analysis using the SEM Framework. A priori domains from the SEM Framework structured the initial analytic matrix; within each domain, we inductively generated subcodes and themes via iterative coding, constant comparison, memo-writing, and team discussion. Specifically, two researchers (one MD, one MA) reviewed accumulated evidence, they were able to recognize emerging themes to develop open codes, and consequently, a matrix was generated in Microsoft Word that included codes, definitions, inclusion/exclusion criteria (i.e., “when to use” and “when not to use” for some variables), and examples from the transcripts [28, 29]. Disagreements in identifying and defining codes were resolved through discussion during weekly team meetings. Once newly refined codes were developed, they were integrated into the codebook to improve code definitions and strengthen the credibility and confirmability of the data. Additionally, an audit trail was maintained to track collected data, memos, meetings, and other key information [30]

After the codebook was formalized, two research personnel (T.P. and M.J.) independently coded the transcripts using MAXQDA software and held weekly meetings to reconcile discrepancies and produce intercoder reliability. They also worked collaboratively to build summaries that would capture apparent impressions and themes regarding several factors, such as partner knowledge of and involvement with their PrEP use, disclosure of their partner’s HIV status and their own PrEP use, the overall experience of taking PrEP in the context of partnerships and types of support received from partners, partnership barriers and facilitators to PrEP adherence, and future intentions to continue using PrEP use. Themes and example excerpts were characterized by age, drug level, and the number of weeks in the study when the sample was collected for drug level testing. TFV-DP concentrations above 700 fmol/punch were considered protective, while those with levels below 700 fmol/punch were not [31]. Potential differences between those with and without protective levels of PrEP (a proxy for adherence) were explored and noted in the description of results.

## Results

### Participant Characteristics

Twenty participants enrolled in the study, with a median age of 37.5 years (Interquartile Range [IQR] 32, 48). Forty-five percent (*n* = 9) self-identified as White, 30% (*n* = 6) as Black, 10% (*n* = 2) as Asian, 10% (*n* = 2) as mixed race, and 5% (*n* = 1) as American Indian/Alaska Native; twenty-five percent (*n* = 5) identified as Hispanic. The median TDF-FTC concentration was 1159 fmol/punch (IQR 689, 1532.5) from the parent study visit prior to their interview, and *n* = 13 (65%) had protective DBS levels (Table 1).

**Table 1:** Characteristics of women in serodiscordant relationships taking PrEP

Participant	Age	Race	Ethnicity	TDF-FTC concentration (fmol/punch)
1	53	White	Not Hispanic	665
2	35	White	Not Hispanic	**1787**
3	38	White	Not Hispanic	**1139**
4	29	White	Hispanic	**1225**
5	53	Asian	Not Hispanic	**4756**
6	45	White	Not Hispanic	**3060**
7	51	White	Not Hispanic	**2792**
8	46	Black	Not Hispanic	636
9	32	Black	Hispanic	**1014**
10	44	Black	Not Hispanic	694
11	32	Asian	Not Hispanic	305
12	37	Black	Not Hispanic	**702**
13	65	Black	Not Hispanic	**1446**
14	31	Native American	Hispanic	**703**
15	43	White	Not Hispanic	**1416**
16	37	Black	Not Hispanic	0
17	26	Native American / White / Black	Not Hispanic	**1636**
18	25	White	Hispanic	674
19	37	Native American / Black / Other	Not Hispanic	**1498**
20	50	White	Hispanic	391

Bold TFV-DP concentrations above 700 fmol/punch were considered protective

### Themes

The following sections describe major themes that explore the role of partner dynamics in cisgender women’s PrEP use, adherence, and intention to continue PrEP. Barriers to and facilitators of PrEP adherence within the context of their partnerships and specific ways partners support PrEP use are also highlighted. Using the SEM framework, the majority of interview content involved interpersonal, community, and structural levels. Thus, for the current article, these three components and their subthemes (interpersonal – imparting knowledge, shared responsibility, childbearing intentions, preventing HIV transmission, mistrust, perceived adverse effects; community – privacy, HIV and PrEP stigma; structural – technological support, PrEP access) are explored in detail.

## Interpersonal

### Imparting Knowledge

Nearly all women disclosed to their partners that they were using PrEP. In fact, several women were introduced to PrEP from their partners, with one noting:

“…As soon as we met, we had a conversation after the third day. Within the month, we had set an appointment to go to the doctor together to talk to his doctor about getting me on PrEP. He already knew about it… He’s the one that educated me about it.” (White, Hispanic, 25 years old, TFV-DP concentration 674 fmol/punch at 24 weeks)

Another participant, who learned about PrEP from her partner’s HIV provider, described how supportive the provider was for her to take PrEP.

“When he was diagnosed and he shared it with me, they [the doctors] wanted me to come in ‘cause they knew that we were in a long-term relationship, and we were trying to have a baby. They wanted me to come in, so when we came and got tested, they told me everything was fine with me. They asked about how I feel doing a study, and of course, I agreed to it because I intend on staying in the relationship, and we want to move forward with starting a family and things like that.” (Black, Hispanic, 32 years old, TFV-DP concentration 1014 fmol/punch at 24 weeks)

Another participant described learning about PrEP from her partner and how appreciative he was when she began taking the medication.

“I would say early on there was definitely a lot more discussion. He basically told me about it, and then he was like, ‘Is this something you would be willing to do?’ and I was like, ‘Of course.’ Once I first started taking it, it was more like, ‘How do you feel?’ He was always checking in with me, and then when we would be taking the pills, he’s very appreciative of the fact that I’m doing this for us because he basically knows I’m taking this thing that, at this point, I don’t feel any bad negative effects, but I don’t know the long-term effects of it.” (White, non-Hispanic, 38 years old, TFV-DP 1139 fmol/punch at 50 weeks)

### Shared Responsibility

Women who reported feeling supported by their partners to take PrEP usually had TFV-DP concentration > 700 fmol/punch. The main forms of partner support described were reminding them to take their medication, as well as taking medications together. One woman recalled how her partner reminded them to take their medications together even if they initially forgot and stated that her partner “…will remember, and he will jump up if he fell asleep, like, ‘Hold on!’ and get up and make sure we take our meds.” (Native American, non-Hispanic, 37 years old, TFV-DP concentration 1498 fmol/punch at 24 weeks). Another participant described the routine she and her partner fell into that helped their mutual adherence. “At this point, it’s rhythm for us. He takes his pill; he gets my pill and my bottle and gives it to me. I take it, and we go to bed, so it’s just really easy and breezy for us.” (White, non-Hispanic, 38 years old, TFV-DP 1139 fmol/punch at 50 weeks).

Nevertheless, support from partners was not always enough for participants with suboptimal levels. One participant shared, “When he’s home with me, I always take my pill at the same time he does ‘cause he’s the one that will remind me. He will push me, you know? He’ll be like, ‘Come on. It’s time.’” (White, Hispanic, 25 years old, TFV-DP concentration 674 fmol/punch at 24 weeks). Despite this participant’s partner being supportive of her PrEP use, her DBS results did not reach protective levels. Across levels of protection, participants acknowledged other ways their partners had positively impacted their PrEP adherence, such as providing words of encouragement, accompanying them to doctor’s appointments, and having discussions about HIV and PrEP.

Alternatively, some women felt that their partners were discouraging of their PrEP use. In response to asking if her partner was supportive of her decision to take PrEP, one participant stated:

“No, he was a little irritated, almost like, ‘Well, you don’t believe me that my numbers are down.’ I said, ‘No, I believe you that your numbers are down. I believe that you probably can’t transfer it to me right now. All you need is to get sick one time, and your numbers will flip.’” (White, non-Hispanic, 43 years old, TFV-DP concentration 1416 fmol/punch at 36 weeks)

### Childbearing Intentions

Significant motivators for taking and remaining adherent to PrEP included the desire to have children and being committed to their partner. Some women talked about the protective effect of PrEP to prevent perinatal HIV transmission. “If we had a kid, it would make it easier if I didn’t have it [HIV]… and also it [PrEP] would be protective for the child as well.” (White, non-Hispanic, 35 years old, TFV-DP level 1787 fmol/punch 18 weeks) Another woman shared, “Like I said, me wanting to have a baby soon… it keeps me motivated to continue to take it.” (Black, Hispanic, 32 years old, TFV-DP concentration 1014 fmol/punch at 24 weeks).

### Preventing HIV Transmission

Women talked about their partners supporting their PrEP use because they did not want them to contract HIV. One participant said, “He wants me to be healthy. He wants me to stay negative and stay in the clear, doesn’t want me to be sick, so that’s the main reason of why he’s in support of it.” (Black, Hispanic, 32 years old, TFV-DP concentration 1014 fmol/punch at 24 weeks).

Some described the acceptance of their partner’s HIV infection as a driving force to take PrEP, with one reporting:

“Just the commitment that I made – I knew this was going to be my life, and so that’s the mindset I have to go in, and that’s what I use to help my husband when he goes into those moments where he’s just discouraged or sad because of how life may affect me because of it [HIV], but it’s our life. It is what it is. I have to take a pill. I want to take a pill every day. My driving force is that it’s just a part of my life.” (Native American, non-Hispanic, 26 years old, TFV-DP concentration 1636 fmol/punch at 12 weeks)

Women often also indicated their commitment to their partner as a major facilitator of their PrEP adherence:

“After waking up, it just became like an extra pill. I don’t really know why all of a sudden I’m so willing to participate in my healthiness… I guess because I found a reason to do it because if I’m around longer and he’s around longer, then we can both be together longer. That’s whatever we got to do to make that happen.” (White, non-Hispanic, 45 years old, TFV-DP concentration 3060 fmol/punch at 8 weeks)

Only one woman shared that she would be comfortable discontinuing PrEP once their partner’s viral load became and remained undetectable, acknowledging the scientific principle of undetectable equals untransmittable (U = U) [32].

“But once he’s there and he’s undetectable and he’s on his regular cycle of meds – because they’re trying to come out with a shot, and while they’re changing stuff, I wanna be protected… but once he’s good, then I’m probably gonna stop taking it.” (Native American, non-Hispanic, 37 years old, TFV-DP 1498 fmol/punch at 24 weeks)

### Mistrust

There were partners who disapproved of the participant’s PrEP use because they perceived it as an issue of distrust or infidelity within their partnership. Rather than viewing PrEP as an additional layer of protection, a partner may have concluded that the participant took PrEP because they did not believe that their HIV viral load was undetectable [18, 33, 34].

For some women, the decision to take PrEP was impacted by the lack of support from their partners. One participant noted that her partner was not supportive of her using PrEP, and when their relationship ended, she discontinued her PrEP use.

“He said he didn’t think that I should be on it, and I’m like, ‘Well, why am I taking extra pills? I already take enough, so why am I taking more stuff and there’s nothing wrong with me? … I stopped taking it, and I went back to taking it again. Now I don’t have a reason to take it ‘cause we’re not together.” (Black, non-Hispanic, 37 years old, TFV-DP concentration 0 fmol/punch at 24 weeks)

### Perceived Adverse Effects

Another way partners may have influenced medication adherence was their concern about potential inconveniences related to a daily oral medication. One participant stated, “Sometimes [he’ll say], ‘Are you sure you want to take that?’ and he was more concerned that something could happen to me while taking PrEP. I like taking PrEP because I just take it every night… he thinks he gives me a lot of responsibility, like burden, and it’s not.” (Native American, Hispanic, 31 years old, TFV-DP concentration 703 fmol/punch at 36 weeks).

These findings underscore the prominent role partner support may play in medication adherence and possible challenges to adherence when there is insufficient support within serodiscordant couples.

## Community

### Privacy

Most women did not disclose their PrEP use to others because they wanted to protect their partner’s privacy. The overall expressed concern was that disclosing their PrEP use would expose their partner’s HIV status. When asked if anyone else knew about their PrEP use besides her partner, one participant responded:

“No, a lot of people I know know my boyfriend, so I feel like if I told them, I would be telling them that he had HIV, so I don’t think that’s appropriate… I think PrEP is incredible, and if you’re single, there’s no reason to hide it. You should tell all your friends to get on PrEP, so no one gets HIV, but if you’re in a relationship, I feel like that is really telling. You’re saying something – a hidden message. It’s not really hidden; everyone knows….I don’t want him to be judged by my friends.” (White, Hispanic, 29 years old, TFV-DP concentration 1225 fmol/punch at 8 weeks)

### HIV Stigma

For many women, perceptions about taking a medication to prevent HIV was also a significant concern. HIV stigma prevented one participant from sharing knowledge about PrEP to those who might have benefitted from this medication:

“I feel like there’s such a stigma about it, and I feel like even if I had people around me that may benefit from it, it’s not something that anybody is willing to share with anyone else. It’s sad because I know there are a lot of young girls that are out here that can really benefit from it, and they have no idea about it because they’re scared to talk to people about their situation.” (Black/Hispanic, 32 years old, TFV-DP concentration 1014 fmol/punch at 24 weeks)

### PrEP Stigma

Discrimination surrounding sex and assumptions about risk were also discussed as possible reasons people may not want to use PrEP, with one noting:

“If I knew about PrEP, I would’ve told them about it before. For a single person, I think the stigma is just around being sexual in general… If you feel weird or you’re uncomfortable with people talking about sex or people having sex freely, not in a monogamous relationship or not when they’re married, then you might be uncomfortable about PrEP. If there’s no stigma around sex, then there shouldn’t be this stigma around PrEP, but I don’t know how I would eliminate that. That’s a lot of work.” (White/Hispanic, 29 years old, TFV-DP concentration 1225 fmol/punch at 8 weeks)

A few participants echoed similar sentiments, expressing concern about the shame they may endure from others knowing they were sexually active. Concern about being labeled as “promiscuous” or experiencing judgment for having multiple partners, discouraged participants from disclosing their PrEP use with others.

## Structural

### Technological Support

For some, the study’s text messages supported PrEP adherence. One woman’s partner used those reminders to maintain both of their medication adherence:

“I get the text message from [research staff name]. He actually gets it. It goes to his phone, so that’s his reminder to make sure we take our pills. It’s his way of caring for me. He was coming with me to all my appointments, but now he goes to school during the day.” (Native American, non-Hispanic, 37 years old, TFV-DP concentration 1498 fmol/punch at 24 weeks).

### PrEP Access

Although several women voiced concerns about the cost of medication, managing side effects, and gaining access to a clinic as potential barriers to future PrEP use, many were either insistent that they would continue PrEP or were already in the process of maintaining continuity of PrEP use when the study ended. When asked if she would continue PrEP, one participant said, “I would like to. I’m hoping this is something that I can take for a lifetime. Obviously, I’ll need it, unless a miracle happens, but hopefully I can continue to take it.” (Black, Hispanic, 32 years old, TFV-DP 1014 fmol/punch at 24 weeks).

Another woman was already in the process to get PrEP prescribed once the study was over:

“I planned well in advance when I knew I would be out of PrEP so that I can say, ‘Here’s all the test results. What do I need to get you guys for your level of comfort? What test do I need to take with you?’ And like just right now, we’ve started the last bottle. Now I’m at that point where I’m like, ‘Okay, I got the test results.’ Text them all the information today. Get in next week, and I still have plenty of time.” (White, non-Hispanic, 38 years old, TFV-DP 1139 fmol/punch at 50 weeks)

Women who intended to continue taking PrEP may have felt better equipped to continue taking PrEP outside of the study after gaining knowledge about the logistics needed to obtain and persist on PrEP.

## Discussion

**Fig. 1:**
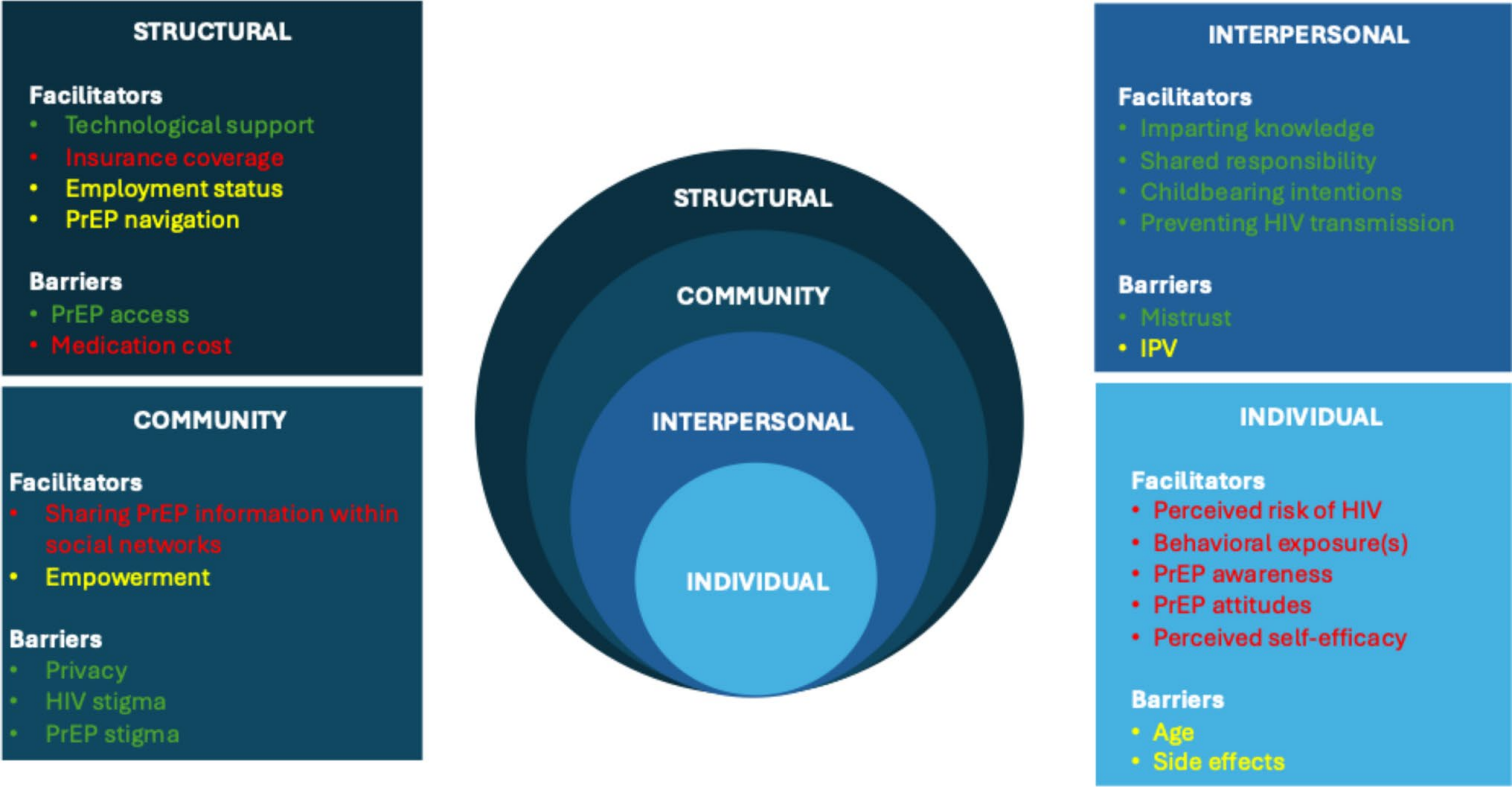
Sociological model of the impact of partner dynamics. The figure shows factors related to partner dynamics that impact PrEP use. Text in green are factors identified in the study. Text in red are aother factors discussed in interviews but not discussed in this analysis. Text in yellow are factors described from literature (Color figure online)

This study extends research exploring barriers to and facilitators of PrEP adherence affecting a sample of cisgender women in serodiscordant relationships with varying PrEP adherence levels. Using a SEM framework, we identified interpersonal, community, and structural facilitators and barriers to PrEP adherence from the perspectives of cisgender women in serodiscordant partnerships in Southern California. This allowed us to develop a SEM demonstrating the impact of partnership dynamics on various levels of PrEP adherence, specifically at the interpersonal, community, and structural levels (Fig. 1). Growing literature on the impact of partner dynamics on PrEP adherence among serodiscordant MSM has led to the recommendation to integrate relationship contexts into HIV prevention programs. Our study findings add to the limited evidence regarding the impact of partner dynamics on PrEP adherence among US-based cisgender women.

Similar to a recent study of women at a U.S. urban family planning health center who identified relationship dynamics as a key contributor to their potential PrEP use [35], we found that partners living with HIV can influence PrEP uptake and adherence for cisgender women. Many participants in our study learned about PrEP from their partners, who were usually informed by their HIV providers. This sharing of information could be described as a form of informational partner support, as these women had no prior knowledge of PrEP and were then presented with a trustworthy strategy to reduce their risk of HIV. Mounting literature indicates that, for some groups, social support plays a pivotal role in increasing PrEP awareness [36]. This strategy may prove to be useful for targeted initiatives to increase PrEP awareness among reproductive-age women in high-risk settings, as PrEP awareness in this group is reportedly low [37].

At the interpersonal level, partner support was found to play a notable role in PrEP adherence, which was displayed through a range of behaviors (e.g., imparting knowledge, sharing responsibility). Some partners would take their HIV medication regimen at the same time as the participants’ PrEP medication, which facilitated support and motivation to continue taking oral PrEP. Partners also provided reminders, and some even administered the participants’ PrEP medication for them. Partners also often verbally expressed their support and appreciation of participants for taking and persisting on PrEP. However, some participants described their partner’s lack of support or discouragement of their PrEP use, which may have impacted their PrEP adherence and whether they wanted to continue using PrEP.

Important community-level barriers to PrEP adherence were stigma and partner privacy. Stigma related to both HIV and PrEP impacted whether participants decided to disclose their PrEP use and share information about PrEP with others who might benefit. Several interviews highlighted concerns about being seen as promiscuous and shamed by their peers and other community members. Additionally, participants detailed how disclosing their PrEP use could result in unintentionally disclosing their partner’s HIV status. Even when participants personally knew someone who could benefit from taking PrEP, the deep-rooted stigma of HIV and the motivation to protect their partner’s privacy prevented them from sharing information. These findings align with recent literature that identified both stigma and relationship dynamics as critical factors influencing potential PrEP use [35].

Structural factors, including technological support and PrEP access, may be influenced by partner dynamics and can contribute to effective PrEP adherence and future persistence. Women found text message communication from the parent study to support not only their PrEP adherence but also their partner’s adherence to ART. A study of Kenyan cisgender pregnant and postpartum women, some in serodiscordant partnerships, found that two-way text messaging could increase PrEP continuation and adherence [38]. PrEP access, including but not limited to insurance coverage and healthcare navigation, are critical components of PrEP engagement [39]. Women in serodiscordant partnerships may benefit from their partner’s experience related to their own healthcare, which may help to facilitate establishing and maintaining PrEP care. Multi-level interventions are likely needed to improve adherence and persistence in PrEP among cisgender women in serodiscordant partnerships.

Taken together, partner support was not monolithic, but rather motivated by a range of underlying goals that shaped how women perceived and responded to this support. For some, adherence encouragement reflected shared responsibility, with partners synchronizing pill-taking or offering reminders as a sign of solidarity. Others emphasized empowerment, affirming women’s autonomy and framing PrEP as self-care beyond HIV prevention. A third orientation was protective or family-centered, where women’s adherence was seen as essential for family stability and health. While sometimes welcomed, this approach could also feel burdensome, suggesting women carry responsibility primarily for others. Finally, some women experienced support as surveillance, where reminders felt more controlling than encouraging. Thus, partner support operated along multiple motivational pathways—shared responsibility, empowerment, protection, and surveillance—and the same behaviors could be experienced as supportive or coercive, depending on the relational context.

## Limitations

This study is not without limitations. First, participants self-selected to interview. As a result, those who chose to participate may have had more positive experiences with PrEP than those who chose not to participate. In addition, this study was nested within a parent study that had additional support, including text message reminders, that could have influenced our findings. Participants were interviewed at different weeks of the study, with some on PrEP for less time than others, which may have influenced their PrEP experiences. Interviews were conducted by the study PI as opposed to an individual taking PrEP or in a serodiscordant relationship, which could have impacted the level of information divulged during the interviews. There were also no specific interview questions regarding intimate partner violence (IPV). Further research on the impact of IPV on PrEP adherence in the context of serodiscordant couples is vital, as previous studies indicate IPV impedes access PrEP services, uptake, and adherence [40, 41] Thematic saturation among distinct subgroups (e.g., adherence, viral suppression among partners) was not evaluated and may have affected lived experiences around PrEP support, which could have impacted our findings and subsequent conclusions regarding facilitators and barriers to PrEP adherence among these women. Lastly, this study was conducted several years ago which could limit its relevance. Research in this field has expanded; however, literature on PrEP adherence for cisgender women in US-based serodiscordant relationships is still scarce.

## Conclusion

Within the context of this sub-study for HIV-negative cisgender women in serodiscordant relationships taking PrEP, we found that their male partners often served an important role in their experiences with PrEP and that relationship dynamics could act as a facilitator or barrier to PrEP adherence at multiple socio-ecological levels. It is critical to further explore partner dynamics, including the impact of IPV, and PrEP adherence specifically among US-based cisgender women in serodiscordant relationships. Such an understanding may promote the development of more robust and targeted supports for women in HIV prevention.

## Data Availability

Data is available by the authors upon request.
